# Acute Physiological Response Variability in Lidia Bulls Following Roping Bull Celebrations: Effects of Age, Previous Participation, and Sampling Period

**DOI:** 10.3390/ani16172739

**Published:** 2026-09-02

**Authors:** Julio Sedeño, Salvador Ruiz, Germán Martín, Juan Carlos Gardón

**Affiliations:** 1 Doctoral School, Catholic University of Valencia-San Vicente Mártir, 46001 Valencia, Spain; julio.sedeno@ucv.es; 2 Department of Physiology, Faculty of Veterinary Medicine, Campus Mare Nostrum, University of Murcia, 30100 Murcia, Spain; 3 Institute for Biomedical Research of Murcia, IMIB-Arrixaca, 30120 Murcia, Spain; 4 Department of Basic Sciences, Faculty of Veterinary and Experimental Sciences, Catholic University of Valencia-San Vicente Mártir, 46001 Valencia, Spain; german.martin@ucv.es; 5 Department of Medicine and Animal Surgery, Catholic University of Valencia-San Vicente Mártir, 46001 Valencia, Spain

**Keywords:** Lidia cattle, Roping Bull celebrations, animal welfare, stress biomarkers, hematological parameters, biochemical parameters, cortisol, acute exercise

## Abstract

Roping Bull celebrations involve prolonged physical activity in Lidia cattle and are associated with marked acute hematological, biochemical, and endocrine responses. Previous research has characterized these physiological changes, but less is known about the factors that may explain inter-individual variability in their magnitude. The present study evaluated whether age, previous participation history, and sampling period influence the PRE–POST physiological response of Lidia bulls. A total of 101 adult Lidia bulls were evaluated immediately before and after participation in a standardized 45 min Roping Bull event. Fifty-three bulls (52.4%) had also been included in a previous study in which the physiological variables evaluated in the present investigation had been reported. Linear mixed-effects models were fitted separately for each biomarker, with Time (PRE vs. POST), age group (AGE), previous participation history (RACES), and sampling period (MOMENT) included as fixed effects, together with prespecified interactions with Time. Individual bull was included as a random intercept to account for the repeated PRE–POST measurements. Participation was associated with significant changes in several hematological, biochemical, and endocrine biomarkers, particularly lactate, creatine kinase, lactate dehydrogenase, leukocyte variables, cortisol, and ACTH. In contrast, AGE, RACES, and MOMENT showed comparatively limited influence on the magnitude of most PRE–POST responses. Selected Time × factor interactions indicated some biomarker-specific inter-individual variability, although these effects were less consistent than the overall Time effect. The present study extends the previous physiological characterization of Lidia bulls by identifying potential factors associated with variability in the acute PRE–POST response. Overall, the physiological response was relatively consistent across age and previous participation categories, although selected interactions indicate that individual and event-related factors may contribute to variation in specific biomarkers. These physiological measurements should not be interpreted in isolation as direct evidence of either preserved or compromised animal welfare.

## Simple Summary

Traditional “Roping Bull” festivities involve prolonged physical activity in Lidia cattle and have raised interest regarding their physiological demands and potential welfare implications. Previous research has characterized the acute hematological, biochemical, and endocrine responses associated with participation in these events. The present study addresses a different question: whether the magnitude of these previously characterized physiological responses varies according to animal- and event-related factors. A total of 101 Lidia bulls were evaluated immediately before and after a standardized 45 min event. Fifty-three (52.4%) bulls had also been included in the previous physiological study. Linear mixed-effects models were used to evaluate the effects of age, previous participation history, and sampling period on PRE–POST responses. Participation was associated with marked changes in several hematological, biochemical, and endocrine biomarkers, whereas the magnitude of most responses was relatively consistent across age and previous participation categories. Selected interactions indicated some inter-individual variability. These findings extend previous descriptive evidence by identifying the factors associated with variability in the acute physiological response. However, physiological biomarkers alone cannot determine whether animal welfare was preserved or compromised.

## 1. Introduction

The Lidia breed (*Bos taurus brachyceros*) is one of the most distinctive cattle populations of the Iberian Peninsula and represents the second largest purebred cattle population registered in Spain. Officially recognized through Royal Decree 60/2001, this breed has been shaped by centuries of selective breeding aimed at preserving behavioral traits associated with reactivity, mobility, and resistance to handling [1,2]. Although primarily distributed throughout Spain, Lidia cattle are also present in Portugal, France, and several Latin American countries [3,4]. Most animals are managed under extensive production systems, characterized by low levels of human contact and large grazing areas [1,3].

Lidia cattle play a central role in numerous traditional festivities throughout Spain. Among these, Roping Bull celebrations (“Toro de Cuerda”) are widely practiced and involve guiding bulls through urban routes using ropes attached to their horns. These events are known by different regional names, including “bou en corda”, “bou capllaçat”, “toro ensogado”, “toro enmaromado”, and “Sokamuturra” [5]. Despite their cultural significance, concerns regarding their implications for animal welfare have increased in recent decades, highlighting the need for objective scientific evidence regarding their physiological consequences.

Physical exercise, handling procedures, transportation, exposure to unfamiliar environments, and intense sensory stimulation can induce measurable physiological responses in cattle. Previous studies have shown that acute physical and psychological challenges may alter hematological profiles, muscle-associated enzymes, metabolic indicators, and endocrine responses associated with activation of the hypothalamic–pituitary–adrenal (HPA) axis [6,7,8]. These biomarkers are widely used to evaluate physiological adaptation to exercise and stress in livestock species, and have been extensively applied in Lidia cattle to characterize responses to traditional handling and exercise protocols [9,10].

Studies performed in fighting bulls after conventional bullfighting events have reported marked increases in lactate, creatine kinase (CK), lactate dehydrogenase (LDH), cortisol, and other indicators of metabolic and muscular exertion [11,12]. However, conventional bullfighting differs substantially from Roping Bull celebrations in duration, management conditions, and the nature of animal–human interactions. Consequently, the physiological responses reported for fighting bulls cannot be directly extrapolated to animals participating in Roping Bull events.

Research conducted by Sedeño et al. [13] provided, to our knowledge, the first physiological characterization of Lidia cattle participating in Roping Bull celebrations by describing the acute hematological, biochemical, and endocrine responses associated with these events. The present study builds directly upon those findings. Of the 101 bulls included in the present study, 53 (52.4%) had also been included in the previous investigation, and the physiological variables measured in these overlapping animals had previously been reported.

The scientific objective of the present study therefore differs from that of the previous publication. Whereas the previous study primarily described the overall PRE–POST physiological changes associated with participation, the present investigation focuses on potential sources of inter-individual variability in these responses. Specifically, we evaluated whether the magnitude of the PRE–POST physiological changes was influenced by predefined animal- and event-related factors, including age (AGE), previous participation history (RACES), and sampling period (MOMENT), using linear mixed-effects models. Consequently, the novelty of the present study lies in the analysis of factors associated with variability in the previously characterized physiological response rather than in providing a new descriptive characterization of the same physiological variables.

We hypothesized that the magnitude of the acute PRE–POST physiological response would differ according to age and previous participation history, reflecting potential differences in physiological reactivity or prior exposure, and that the sampling period would contribute to variability in selected biomarkers because of potential diurnal effects.

Therefore, the objectives of this study were: (1) to evaluate whether acute PRE–POST physiological responses previously characterized in Lidia bulls vary according to age, previous participation history, and sampling period; and (2) to identify potential sources of inter-individual variability in hematological, biochemical, and endocrine responses using an independent mixed-effects statistical framework.

## 2. Materials and Methods

### 2.1. Ethics

All experimental procedures complied with European Directive 2010/63/EU on the protection of animals used for scientific purposes and Spanish Royal Decree 53/2013 governing animal experimentation. The study protocol was reviewed and approved by the Animal Ethics Committee of the Catholic University of Valencia San Vicente Mártir (approval No. CEEAUCV2203). In addition, written authorization was obtained from the owners of the privately owned bulls for their animals to participate in the study and for blood sampling to be performed. All sampling procedures were conducted by licensed veterinarians experienced in the management of Lidia cattle, and every effort was made to minimize stress and discomfort during animal handling and blood collection.

### 2.2. Animals

#### 2.2.1. Study Population

The study included purebred Lidia bulls originating from officially registered breeding farms included in the Spanish Lidia Herd Book (Libro Genealógico de la Raza Bovina de Lidia). Animals were raised under the traditional extensive production system characteristic of this breed, with permanent access to pasture and limited human contact before participation in the events. A total of 101 Lidia bulls were included in the study. Each bull contributed data from one Roping Bull event only; therefore, each animal provided one PRE–POST observation pair for the present analysis. The animals represented four Spanish autonomous communities: Valencia (*n* = 65), Andalusia (*n* = 25), Castilla-La Mancha (*n* = 6), and Aragón (*n* = 5).

All bulls participated in officially sanctioned continuous-running Roping Bull celebrations organized under the regulations established by the Spanish Federation of the Roping Bull. Of the 101 bulls included in the present study, 53 (52.4%) had also been included in a previous investigation [13]. The physiological variables measured in these overlapping animals had previously been reported. The present study uses these physiological measurements to address a different scientific question, namely, whether the magnitude of the PRE–POST responses varies according to predefined animal- and event-related factors using a linear mixed-effects modelling framework.

#### 2.2.2. Inclusion and Exclusion Criteria

Eligible animals fulfilled the following criteria: (i) confirmed registration in the official Lidia Herd Book; (ii) male sex and an age of at least four years; (iii) satisfactory health certification before the event, including routine sanitary controls; (iv) absence of clinical abnormalities during the veterinary examination performed immediately before participation; and (v) no recent pharmacological treatment that could interfere with hematological, biochemical or endocrine measurements.

Animals were excluded when they presented evidence of disease or injury, incomplete identification records, prolonged transportation immediately before the event, or when blood samples of sufficient quality could not be obtained.

#### 2.2.3. Sample Size and Characteristics

A total of 101 adult Lidia bulls between four and nine years of age were enrolled. Animals weighed between 500 and 540 kg and differed in their previous participation history, which was classified into three categories (0, 1–5 and 5–10 previous participations). Events were conducted either in the morning or in the afternoon. The sampling period was therefore recorded as MOMENT (morning vs. afternoon) and subsequently included as a fixed effect in the statistical models.

### 2.3. Experimental Design and Exercise Protocol

The investigation followed a repeated-measures design in which each bull was sampled immediately before and after participation in a traditional Roping Bull celebration. The Roping Bull activity lasted 45 min according to the established event format and involved prolonged physical activity along the established urban course, with animals guided by participants using the customary rope-handling procedure. This duration should be interpreted as the standardized duration of the event rather than as an indicator of identical individual exercise intensity. Although the event involved sustained locomotor activity, individual running speed, distance travelled, locomotor activity, and periods of movement and rest were not objectively quantified, and the level and pattern of exertion may therefore have varied among individual animals. Accordingly, the present study refers to the resulting physiological changes as an acute PRE–POST response to event participation rather than assuming a uniform exercise intensity across animals. Throughout the celebration, animals followed the established urban course while being guided by participants using the customary rope-handling procedure.

Before the beginning of the event, bulls remained at the celebration site under standardized management conditions to reduce the influence of transport and environmental adaptation. Feed was withheld for approximately 3 h before blood sampling to minimize postprandial variation in circulating biochemical and metabolic variables. Water remained available during this period. A veterinary examination was performed before participation to confirm that all animals met the inclusion criteria.

Blood samples were collected from the coccygeal vein by licensed veterinarians experienced in the handling of Lidia cattle. PRE samples were collected approximately 5 min before the beginning of the event, and POST samples were obtained within approximately 5 min after completion. Sampling was performed under the established management conditions of each event, with the animals handled by experienced veterinary personnel. Shade was available during blood sampling, including during afternoon events.

### 2.4. Sample Collection and Laboratory Processing

#### 2.4.1. Blood Collection

Blood was obtained from the coccygeal vein using sterile 18-gauge needles connected to a vacuum collection system (BD Vacutainer^®^, Becton Dickinson, Franklin Lakes, NJ, USA). Before venipuncture, the sampling area was disinfected with 70% isopropyl alcohol following standard aseptic procedures. Blood was subsequently collected into the corresponding tubes for hematological, biochemical, and endocrine analyses, following the sampling sequence used during the study.

At each sampling time (PRE and POST), three 5 mL blood tubes were collected from every animal. Two K3-EDTA tubes were reserved for hematological and hormonal determinations, whereas one sodium fluoride/potassium oxalate tube was used for lactate analysis. During sequential blood collection, the EDTA-containing tubes were filled before the sodium fluoride/potassium oxalate tube. Blood collection was completed as rapidly as possible in order to minimize handling time and avoid additional stress-related physiological changes.

#### 2.4.2. Sample Processing and Storage Protocols

Immediately after collection, EDTA tubes intended for hematological evaluation were gently mixed by inversion and transported under refrigerated conditions (4 °C). Complete blood counts were performed within two hours of sampling.

Samples designated for biochemical and endocrine analyses were centrifuged within 30 min after collection at 2200× *g* for 10 min at 4 °C using a refrigerated centrifuge (Eppendorf 5810R, Hamburg, Germany). Following centrifugation, plasma was carefully aspirated without disturbing the buffy coat and transferred into sterile polypropylene cryovials identified with the corresponding animal and sampling time.

Plasma aliquots were maintained at 4 °C until laboratory arrival and subsequently stored at −20 °C for short-term preservation. Within the following 24 h, all samples were transferred to a −80 °C freezer, where they remained until analysis. To preserve sample integrity, each aliquot was thawed only once before laboratory determinations.

#### 2.4.3. Quality Control Measures

All specimens were visually inspected before analysis to detect hemolysis, clot formation, or other alterations that could compromise analytical quality. Cold-chain conditions were maintained throughout transport and storage, and sample identification was verified at every processing stage. Laboratory equipment was calibrated according to the manufacturers’ recommendations before sample processing, and all procedures were carried out following standardized operating protocols to ensure consistency throughout the study.

### 2.5. Sample Analyses

#### 2.5.1. Hematology Analysis

Whole-blood samples collected in EDTA were used for complete blood count determination with an automated five-part differential hematology analyzer (Cell-Dyn 3700, Abbott Diagnostics, Abbott Park, IL, USA), according to the laboratory’s established analytical procedures.

The hematological profile included total leukocyte (WBC) and erythrocyte (RBC) counts, platelet count (PLT), hematocrit (HCT), hemoglobin concentration (HGB), and the erythrocyte indices mean corpuscular volume (MCV), mean corpuscular hemoglobin (MCH), and mean corpuscular hemoglobin concentration (MCHC). Differential leukocyte counts (neutrophils, lymphocytes, monocytes, eosinophils, and basophils) were automatically generated by the instrument.

To ensure analytical reliability, samples were analyzed in duplicate and internal quality-control materials supplied by the manufacturer were processed daily according to the laboratory quality assurance protocol.

#### 2.5.2. Biochemistry and Hormonal Analysis

Plasma activities of creatine kinase (CK) and lactate dehydrogenase (LDH) were determined using an automated clinical chemistry analyzer (BA400, Biosystems S.A., Barcelona, Spain) following the manufacturer’s analytical procedures. Both enzymes were quantified by kinetic spectrophotometric methods, and the results were expressed as international units per liter (IU/L).

Plasma lactate concentration was measured using an enzymatic colorimetric assay based on lactate oxidase methodology, with results expressed in mmol/L according to the manufacturer’s calibration standards.

Endocrine biomarkers were analyzed using the IMMULITE 1000 automated immunoassay platform (Siemens Healthineers, Erlangen, Germany). Plasma cortisol concentrations were quantified by a competitive chemiluminescent enzyme immunoassay, whereas adrenocorticotropic hormone (ACTH) concentrations were determined using a two-site sandwich immunoassay according to the manufacturer’s analytical procedure.

All biochemical and hormonal determinations were performed according to the manufacturers’ instructions. Calibration and internal quality-control procedures were carried out before each analytical batch to verify instrument performance and analytical precision. Whenever duplicate measurements exceeded the predefined acceptance limits, the corresponding samples were reanalyzed.

### 2.6. Quality Assurance and Method Validation

Quality assurance procedures were implemented throughout all stages of sample collection, processing, storage, and laboratory analysis to ensure the reliability of the generated data. Analytical instruments were calibrated according to the manufacturers’ recommendations before each series of measurements, and commercial control materials were included routinely to verify instrument performance.

Sample handling followed standardized laboratory procedures designed to preserve specimen integrity. The interval between blood collection and processing was minimized, refrigerated conditions were maintained during transport, and plasma aliquots were stored under controlled temperatures until analysis. Samples showing visible hemolysis, clotting, or any other condition that could compromise analytical accuracy were excluded from further evaluation.

Laboratory analyses were performed by trained personnel using established analytical methods, and all quality-control records were reviewed before the statistical analysis was initiated.

### 2.7. Statistical Analysis

#### 2.7.1. Software and Data Management

Statistical analyses were performed using IBM SPSS Statistics for Windows, version 31.0.1.0 (IBM Corp., Armonk, NY, USA), with a two-tailed α level of 0.05.

Continuous variables are presented as mean ± standard deviation (SD) when normally distributed, and as median and interquartile range (IQR) when normality assumptions were not met. Categorical variables are summarized as frequencies and percentages.

The distribution of each biomarker was assessed using the Shapiro–Wilk test together with visual inspection of histograms and Q–Q plots. When the assumption of normality was violated (Shapiro–Wilk *p* < 0.05 in PRE or POST), a logarithmic transformation (log_10_[x + 1]) was applied to improve the normality of the residuals in the subsequent models.

To evaluate the effects of time and experimental factors on biomarker responses, linear mixed-effects models (LMMs) were fitted separately for each dependent variable. The fixed-effect structure included Time (PRE vs. POST), previous event exposure (RACES; 3 levels), age group (AGE; 3 levels), and measurement moment (MOMENT; 2 levels), as well as the prespecified two-way interactions Time × RACES, Time × AGE, and Time × MOMENT. Higher-order interactions were considered only when biologically justified and supported by the data structure. Subject identification was included as a random intercept to account for within-subject correlation arising from the repeated PRE–POST measurements. Model parameters were estimated using restricted maximum likelihood (REML), and degrees of freedom were computed using the Satterthwaite approximation. Because only two repeated measurements were obtained for each bull (PRE and POST), the covariance structure was specified as compound symmetry (CS). With only two time points, CS provides a single within-subject covariance parameter, and the distinction between CS and an unstructured covariance specification is limited. The random-intercept formulation was therefore considered appropriate for accounting for within-subject correlation.

For each fixed effect, the Wald chi-squared statistic (χ^2^), degrees of freedom, *p*-value, and partial eta-squared (η^2^p) effect size were reported. η^2^p was interpreted following Cohen’s conventions: <0.01, trivial; 0.01–0.06, small; 0.06–0.14, medium; 0.14–0.26, large; and ≥0.26, very large. Effect sizes were expressed as partial eta-squared derived from Wald χ^2^ statistics to facilitate comparison across biomarkers. Although alternative measures such as marginal and conditional R^2^ have been proposed for mixed-effects models, η^2^p was retained because the primary objective was to quantify the relative magnitude of the Time effect for each biomarker.

When significant main effects of Time or statistically meaningful interactions were detected, post hoc pairwise comparisons were performed using estimated marginal means (EMMs). Bonferroni adjustment was applied to these post hoc comparisons to control for multiple testing within each family of pairwise contrasts. For the most relevant pairwise contrasts (PRE vs. POST within each level of a significant moderator), Cohen’s d for paired samples and its 95% confidence interval were computed and reported alongside the adjusted *p*-values.

Model assumptions were evaluated through inspection of residual plots, including assessment of homoscedasticity (residuals vs. predicted values) and normality of residuals (Q–Q plots and histograms of residuals). Extreme values were flagged using standardized residuals (|z| > 3). No data imputation was performed; analyses were conducted on complete cases.

#### 2.7.2. Assessment of Multicollinearity Between Experimental Factors

Before fitting the linear mixed-effects models, the potential association and multicollinearity between the experimental factors AGE and RACES were assessed. Because both factors were categorical with three levels, a contingency table analysis was conducted, and independence was evaluated using Pearson’s chi-squared test and Cramér’s V. Spearman’s correlation was also calculated as a complementary measure of association. Multicollinearity was further assessed using variance inflation factors (VIF) calculated for the corresponding dummy-coded levels of AGE and RACES.

No statistically significant association was observed between AGE and RACES (χ^2^ = 0.668, df = 4, *p* = 0.955; Cramér’s V = 0.058), and Spearman’s correlation was negligible and non-significant (ρ = 0.071, *p* = 0.481). All VIF values were <1.11, indicating no evidence of relevant multicollinearity and supporting the simultaneous inclusion of AGE and RACES as fixed effects in the LMMs.

#### 2.7.3. Sample Size and Statistical Power

A priori power analysis was conducted using G*Power 3.1 [14] for a two-tailed paired-samples design. With *n* = 101, the study had 80% power to detect a standardized effect size of Cohen’s dz ≥ 0.28 at α = 0.05. This effect size threshold is below the smallest effect of interest considered for the primary PRE–POST physiological comparisons, indicating that the sample size was adequate to detect small-to-moderate acute physiological changes.

#### 2.7.4. Multiple Testing and Family-Wise Error Control

Because multiple biomarkers and moderator effects were evaluated in this study, special consideration was given to the interpretation of statistical significance and control of family-wise error. The linear mixed-effects models (LMMs) constituted the primary inferential framework of the study. Therefore, multiplicity correction was not applied to the primary Wald test of the Time effect across the 18 biomarkers, consistent with the hypothesis-generating objective of evaluating multiple, physiologically related biomarkers.

For secondary analyses, Bonferroni adjustment was applied to post hoc pairwise comparisons derived from significant effects within each LMM. The prespecified two-way interaction terms (Time × RACES, Time × AGE, and Time × MOMENT) were evaluated within each biomarker, with interpretation based on the adjusted significance threshold specified for the corresponding family of tests. Higher-order interactions, when retained because they were biologically justified and supported by the data structure, were considered exploratory and interpreted cautiously.

The Bonferroni procedure was selected because it provides a conservative control of the family-wise error rate for multiple pairwise comparisons while maintaining transparent interpretation of the results. Accordingly, statistically significant findings from secondary interaction and post hoc analyses were interpreted in conjunction with effect sizes and confidence intervals rather than based on *p*-values alone.

## 3. Results

### 3.1. Sample and Baseline Characteristics

A total of 101 adult male Lidia bulls (RACES distribution: 55/24/22 for levels 1/2/3; AGE distribution: 56/23/22 for 4–5/6–7/8–9 years; MOMENT distribution: 60/41 for level 0/1) contributed complete PRE and POST measurements for all 18 hematological, biochemical, and hormonal biomarkers. No missing values were observed for any of the study variables. Descriptive statistics (mean ± SD or median [IQR]) for each biomarker at the PRE and POST assessments are reported in Table 1; the normality assessment and the resulting log_10_(x + 1) transformation decisions are summarized in Table 2.

### 3.2. Main Effect of Time on Biomarker Responses

**Figure 1:**
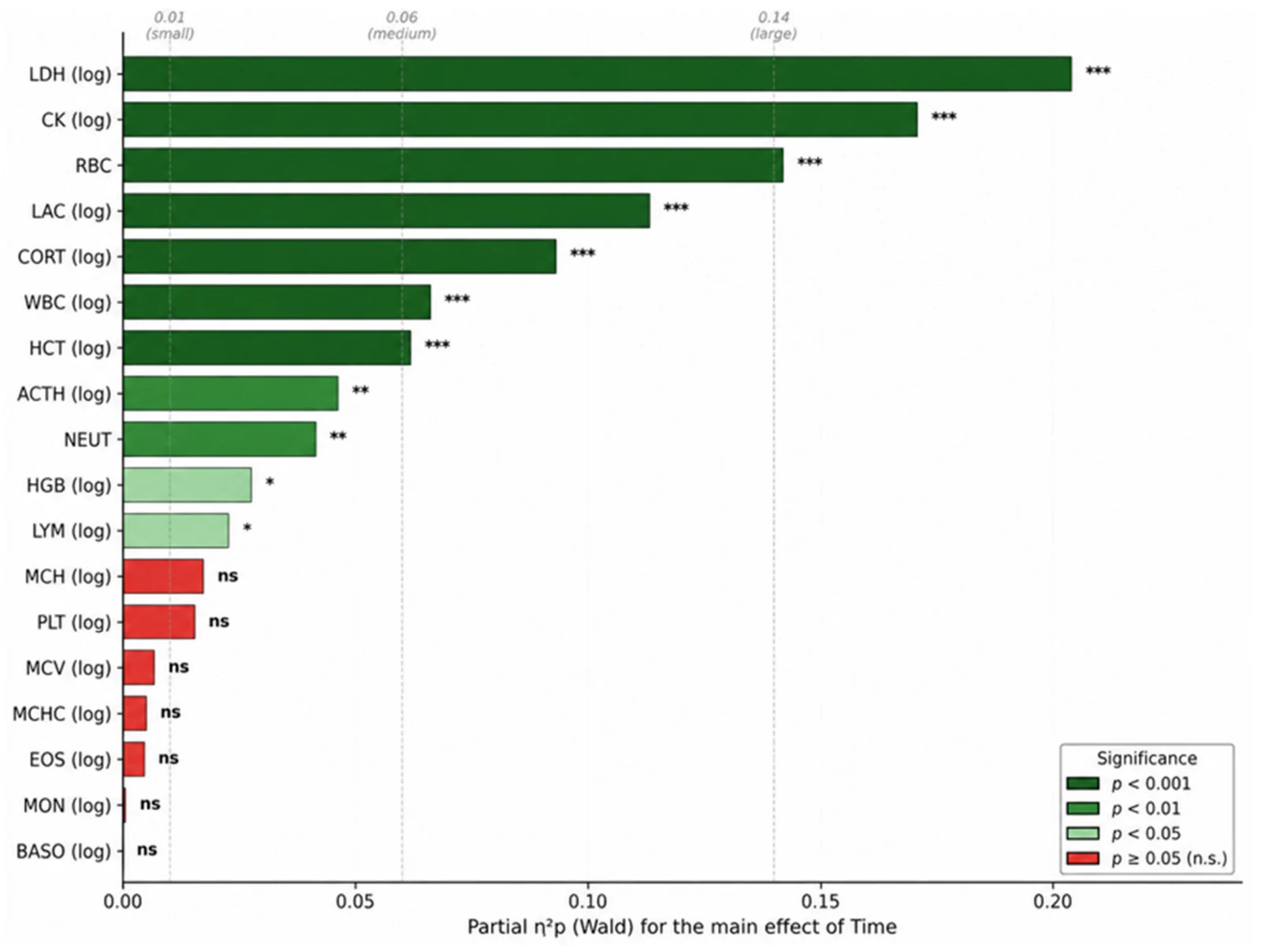
Partial eta-squared (η^2^p) for the main effect of Time (PRE vs. POST) on each of the 18 biomarkers. * Significance levels are indicated by asterisks: * *p* < 0.05, ** *p* < 0.01, and *** *p* < 0.001. “ns” indicates a non-significant result (*p* ≥ 0.05).

The LMM analyses revealed a statistically significant main effect of Time (PRE vs. POST) for 11 of the 18 biomarkers (Table 3, Figure 1). The magnitude of the Time effect, quantified by partial eta-squared (η^2^p), ranged from small to very large across these variables:

- Large effect (0.14 ≤ ηp < 0.26): LDH (ηp = 0.204, *p* < 0.001), CK (ηp = 0.171, *p* < 0.001), and RBC (ηp = 0.142, *p* < 0.001).
- Medium effect (0.06 ≤ ηp < 0.14): LAC (ηp = 0.113, *p* < 0.001), CORT (ηp = 0.093, *p* < 0.001), WBC (ηp = 0.066, *p* < 0.001), and HCT (ηp = 0.062, *p* < 0.001).
- Small effect (0.01 ≤ ηp < 0.06): ACTH (ηp = 0.046, *p* = 0.005), NEUT (ηp = 0.041, *p* = 0.007), HGB (ηp = 0.028, *p* = 0.030), and LYM (ηp = 0.023, *p* = 0.045).
- Non-significant (*p* ≥ 0.05; ηp < 0.02): PLT (ηp = 0.015, *p* = 0.107), MCH (ηp = 0.017, *p* = 0.088), MCV (ηp = 0.007, *p* = 0.288), MCHC (ηp = 0.005, *p* = 0.362), EOS (ηp = 0.005, *p* = 0.384), MON (ηp = 0.001, *p* = 0.768), and BASO (ηp < 0.001, *p* = 1.000). BASO showed essentially no variance in the POST measurement (all values = 0) and is therefore reported but not interpreted.

**Table 3:** Summary of the linear mixed-effects model results for the main effect of Time and the two-way Time × Factor interactions, including partial eta-squared (η^2^p) effect sizes.

Biomarker	Transform	η^2^p (Time)	Wald χ^2^ (Time)	df (Time)	*p* (Time)	*p* (T × R)	*p* (T × A)	*p* (T × M)	Other Significant Interactions
WBC	log_10_	0.066	7.07	1	<0.001	0.001	0.431	0.501	R × A, T × A, T × R × A, T × A × M, T × R × A × M
RBC	—	0.142	16.55	1	<0.001	0.211	0.111	0.170	—
PLT	log_10_	0.015	1.52	1	0.107	0.459	0.225	0.203	—
HCT	log_10_	0.062	6.61	1	0.001	0.406	0.022	0.270	R × M, T × A, T × R × A
HGB	log_10_	0.028	2.88	1	0.030	0.214	0.225	0.203	R × M
MCV	log_10_	0.007	0.70	1	0.288	0.025	0.002	0.434	T × A, T × R × A, T × A × M
MCH	log_10_	0.017	1.73	1	0.088	0.661	0.275	0.291	—
MCHC	log_10_	0.005	0.50	1	0.362	0.812	0.478	0.013	M, T × M
NEUT	—	0.041	4.28	1	0.007	0.802	0.175	0.076	—
EOS	log_10_	0.005	0.50	1	0.384	0.619	0.166	0.193	R × A, A × M
BASO	log_10_	0.000	0.00	1	1.000	1.000	0.633	0.999	T × R × M
LYM	log_10_	0.023	2.35	1	0.049	0.792	0.117	0.042	—
MON	log_10_	0.001	0.10	1	0.768	0.538	0.340	0.249	—
LDH	log_10_	0.204	25.63	1	<0.001	0.831	0.197	0.184	—
CK	log_10_	0.171	20.63	1	<0.001	0.291	0.190	0.159	—
LAC	log_10_	0.113	12.74	1	<0.001	0.291	0.136	0.260	R × A
CORT	log_10_	0.093	10.25	1	<0.001	0.601	0.369	0.264	—
ACTH	log_10_	0.046	4.82	1	0.005	0.562	0.335	0.320	R × A, A, R × A × M

Footnote: Summary of the linear mixed-effects model results. The Wald χ^2^ statistic and degrees of freedom (df) are reported for the main effect of Time (PRE vs. POST) on each biomarker. Partial eta-squared (η^2^p) is reported as a standardized measure of effect magnitude. Effect-size interpretation followed Cohen’s conventions: <0.01 trivial, 0.01–0.06 small, 0.06–0.14 medium, 0.14–0.26 large, and ≥0.26 very large. *p*-values correspond to unadjusted model-based tests from the linear mixed-effects models. Bonferroni correction was applied only to post hoc pairwise comparisons based on estimated marginal means (reported in Table 4). T = Time; R = RACES; A = AGE; M = MOMENT. Higher-order interactions with statistically significant Wald χ^2^ tests are reported in the rightmost column and should be interpreted as exploratory, given the limited statistical power for complex interaction effects. Lactate concentrations are presented in mg/dL; to convert to mmol/L, values should be multiplied by 0.111.

**Table 4:** Post hoc pairwise PRE vs. POST comparisons (Bonferroni-adjusted) for the biomarkers with a significant main effect of Time, with Cohen’s d for paired samples and 95% confidence intervals.

Biomarker	Mean Change (PRE → POST)	Cohen’s d [95% CI]	*p* (Bonferroni)
WBC	+9.5 × 10^3^/µL	0.80 [0.56, 1.04]	<0.001
RBC	+0.5 × 10^6^/µL	1.22 [0.95, 1.49]	<0.001
HCT	+2.0%	0.73 [0.49, 0.97]	<0.001
HGB	+0.7 g/dL	0.66 [0.43, 0.89]	<0.001
NEUT	+3.7%	0.62 [0.39, 0.85]	<0.001
LYM	−3.2%	−0.53 [−0.76, −0.30]	<0.001
LDH	+218.5 IU/L	1.34 [1.06, 1.62]	<0.001
CK	+81.5 IU/L	1.02 [0.76, 1.28]	<0.001
LAC	+8.3 mg/dL	0.91 [0.66, 1.16]	<0.001
CORT	+1.08 µg/dL	0.73 [0.49, 0.97]	<0.001
ACTH	+3.8 pg/mL	0.51 [0.28, 0.74]	0.005

Footnote: Post hoc pairwise comparisons were performed using estimated marginal means with Bonferroni adjustment within each family of pairwise contrasts. Cohen’s d for paired samples was computed as the mean of the PRE–POST differences divided by the standard deviation of the differences. 95% confidence intervals were derived from the t-distribution. WBC = white blood cells; RBC = red blood cells; HCT = hematocrit; HGB = hemoglobin; NEUT = neutrophils; LYM = lymphocytes; LDH = lactate dehydrogenase; CK = creatine kinase; LAC = lactate; CORT = cortisol; ACTH = adrenocorticotropic hormone. Negative values for LYM reflect the expected stress-associated lymphocytopenia. Lactate concentrations are presented in mg/dL; to convert to mmol/L, values should be multiplied by 0.111.

**Figure 2:**
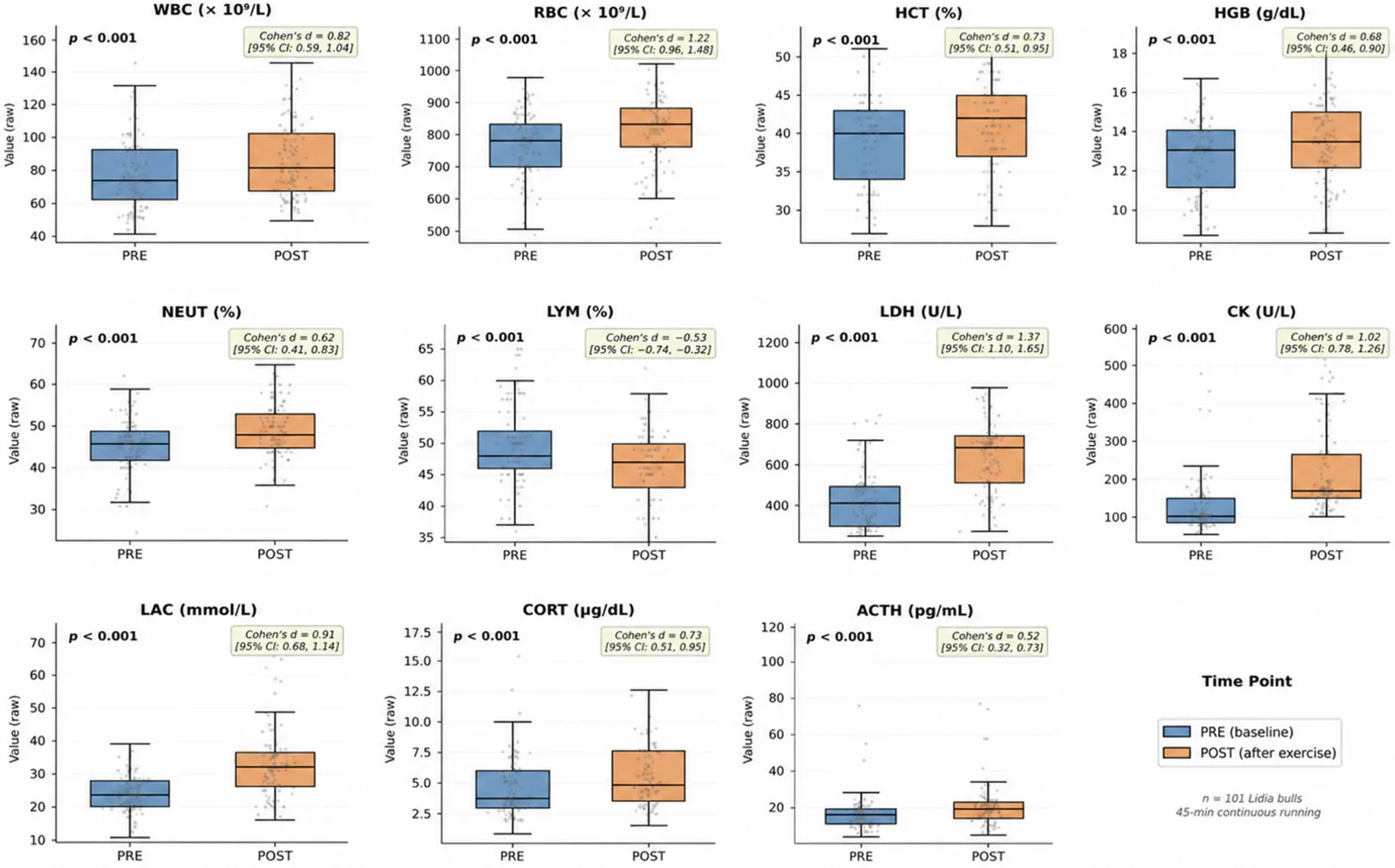
Distribution of PRE and POST values for the eleven biomarkers showing a significant main effect of Time in the linear mixed-effects model (*n* = 101 bulls). Box plots show the median, interquartile range (IQR), and 1.5 × IQR whiskers; grey dots represent individual observations. Values are displayed on the original measurement scale for clinical interpretation. Statistical annotations indicate the *p*-value associated with the main effect of Time in the linear mixed-effects model. Biomarkers are arranged according to the magnitude of the Time effect (η^2^p), encompassing muscle damage (LDH, CK), metabolic response (LAC), endocrine activation (CORT, ACTH), stress-associated leukocyte changes (WBC, NEUT, LYM), and hematological changes (RBC, HCT, HGB).

Overall, the LMM analyses confirmed that 11 of the 18 biomarkers showed a statistically significant Time effect, with magnitudes ranging from small (ηp = 0.023 for LYM) to large (ηp = 0.204 for LDH). The direction of change was consistent with a physiological response to acute exercise: biomarkers of muscular damage (LDH, CK), metabolic stress (LAC), and stress-axis activation (CORT, ACTH) all increased markedly from PRE to POST, alongside a stress-associated leukocyte response (WBC, NEUT) and a response associated with changes compatible with hemoconcentration (RBC, HCT, HGB). LYM decreased, in line with the typical stress-associated lymphocytopenia observed after intense physical exertion. The full distribution of the 11 biomarkers at PRE and POST is shown in Figure 2.

### 3.3. Main Effects of RACES, AGE, and MOMENT

A significant main effect of RACES was observed for WBC (*p* = 0.009) and CORT (*p* < 0.001), indicating that previous participation history was associated with differences in baseline physiological status. A significant main effect of AGE was observed for ACTH (*p* = 0.009) and for several additional stress-axis and red-cell biomarkers. No significant main effects of MOMENT were detected for any of the 18 biomarkers (all *p* > 0.05), suggesting that the diurnal timing of the event did not substantially affect the measured physiological parameters.

### 3.4. Time × Experimental Factor Interactions

The two-way interactions Time × RACES, Time × AGE, and Time × MOMENT were systematically tested. Significant interaction effects were identified for WBC (Time × RACES), HCT (Time × AGE), MCV (Time × RACES and Time × AGE), MCHC (Time × MOMENT), and LYM (Time × MOMENT) (Table 3). Post hoc comparisons were subsequently performed using Bonferroni-adjusted estimated marginal means.

- Time × RACES for WBC (Wald χ^2^ = 12.48, df = 2, *p* = 0.001): the magnitude of the PRE→POST change in leukocyte count differed across previous-experience strata. Post hoc comparisons with Bonferroni correction indicated that the Time effect remained statistically significant in all three RACES strata (*p* < 0.05), with a larger effect size in animals with more previous entries (5–10).
- Time × AGE for HCT (Wald χ^2^ = 7.71, df = 2, *p* = 0.022): a parallel pattern: the magnitude of the PRE→POST increase in hematocrit was larger in younger bulls (≤5 years).
- Time × MOMENT for MCHC (Wald χ^2^ = 6.23, df = 1, *p* = 0.013): the small PRE→POST difference in MCHC was observed only in one of the two sampling moments (morning vs. afternoon).
- Time × RACES for MCV (Wald χ^2^ = 7.37, df = 2, *p* = 0.025): the PRE→POST change in mean corpuscular volume differed across previous-experience strata, with the largest decrease observed in animals with the most prior entries.
- Time × AGE for MCV (Wald χ^2^ = 12.31, df = 2, *p* = 0.002): a parallel age-related pattern, indicating a greater response associated with changes in erythrocyte indices in younger bulls.
- Time × MOMENT for LYM (Wald χ^2^ = 4.16, df = 1, *p* = 0.042): the response associated with a decrease in circulating lymphocytes was more pronounced in the morning sample.

**Figure 3:**
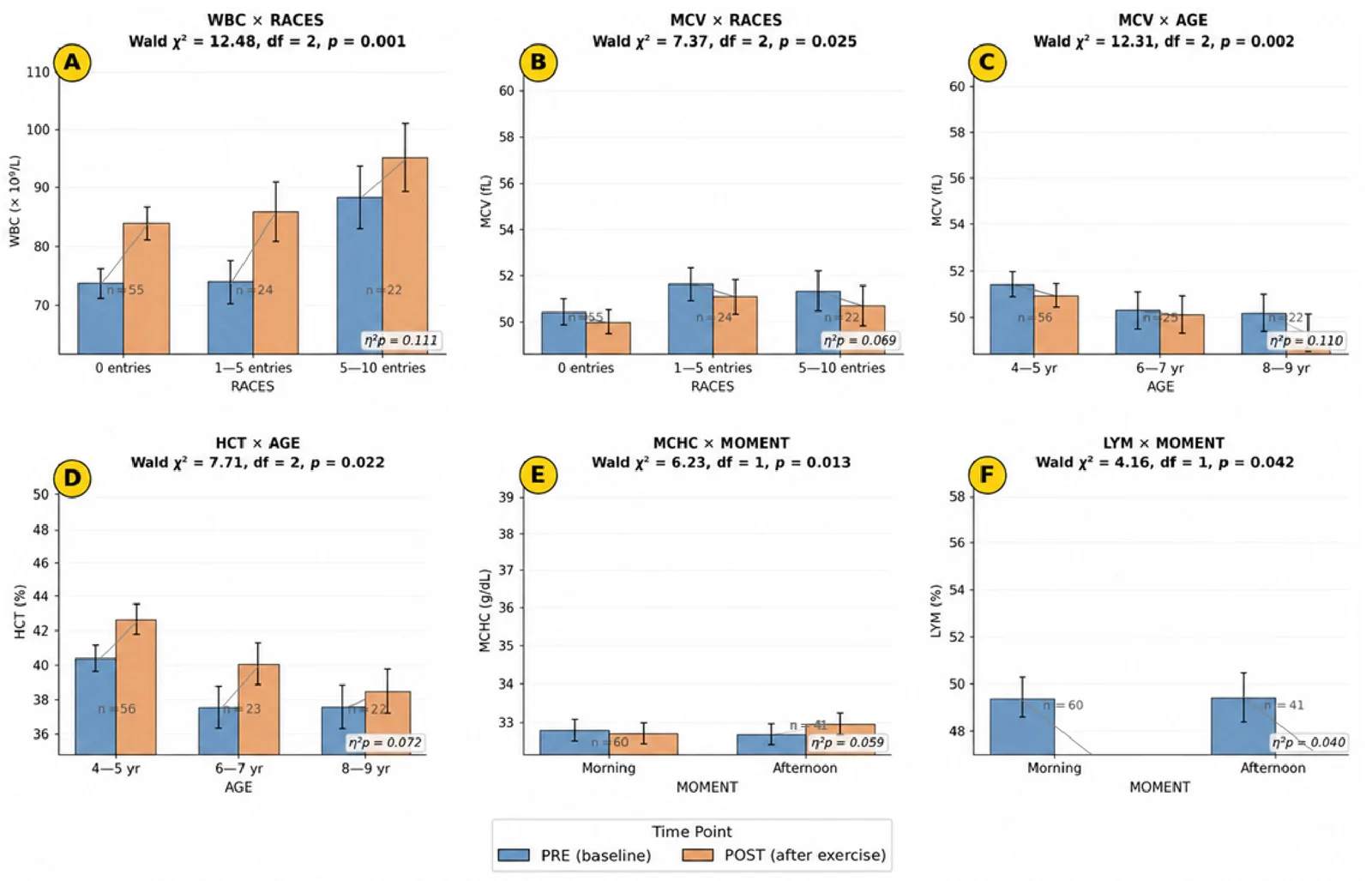
Statistically significant two-way Time × experimental factor interactions were identified in the linear mixed-effects models. Post hoc comparisons were Bonferroni-adjusted where applicable. Bar plots show estimated marginal means ± SEM for PRE (blue) and POST (orange) by level of the moderator factor (RACES, AGE, or MOMENT). Sloped connectors indicate the Time effect within each level; error bars represent SEM. Effect sizes (η^2^p) are reported in the bottom-right of each panel. Panel (**A**): WBC × RACES (Wald χ^2^ = 12.48, df = 2, *p* = 0.001): the PRE→POST change in WBC differed across previous-participation strata; this interaction should be interpreted cautiously because baseline PRE differences may contribute to the observed pattern. Panel (**B**): MCV × RACES (Wald χ^2^ = 7.37, df = 2, *p* = 0.025) and Panel (**C**): MCV × AGE (Wald χ^2^ = 12.31, df = 2, *p* = 0.002): age- and experience-related modulation of response associated with erythrocyte changes. Panel (**D**): HCT × AGE (Wald χ^2^ = 7.71, df = 2, *p* = 0.022): parallel pattern. Panel (**E**): MCHC × MOMENT (Wald χ^2^ = 6.23, df = 1, *p* = 0.013). Panel (**F**): LYM × MOMENT (Wald χ^2^ = 4.16, df = 1, *p* = 0.042).

All other Time × Factor interactions failed to reach statistical significance (*p* > 0.05), indicating that PRE→POST changes were largely homogeneous across the levels of RACES, AGE, and MOMENT. Three-way and four-way interactions that appeared nominally significant (e.g., for WBC, η^2^p = 0.07 for Time × RACES × AGE) are reported as exploratory and should be interpreted with caution, given the limited sample size for the estimation of higher-order interactions. These higher-order interactions were not used to draw substantive biological conclusions regarding age-, previous-participation-, or sampling-period-dependent physiological responses. Figure 3 displays the six statistically significant two-way Time × factor interactions identified in the linear mixed-effects models.

### 3.5. Post Hoc Pairwise Comparisons

For the biomarkers with a significant main effect of Time, the post hoc estimated marginal means (Bonferroni-adjusted) are reported in Table 4. The mean PRE→POST changes in the original measurement scale (back-transformed where applicable) and the associated Cohen’s d effect sizes with 95% confidence intervals indicate very large standardized effects based on Cohen’s d for the muscle-associated and stress-axis biomarkers, and large effects for the hematological markers.

### 3.6. Sensitivity Analysis

To assess the robustness of the main findings to the modelling framework, a sensitivity analysis was conducted using a Generalized Linear Model (GZLM) on the change scores (Δ = POST − PRE), with RACES, AGE, and MOMENT as fixed factors and the PRE value of each biomarker as a covariate. The GZLM analysis confirmed the main effects of Time for the same set of biomarkers identified by the primary LMM (all *p* < 0.05) and yielded qualitatively identical conclusions regarding the between-subject moderators. Full results of the sensitivity analysis are provided in Supplementary Table S1.

### 3.7. Model Assumptions

Inspection of the residual diagnostics did not reveal any substantial violation of model assumptions. Q–Q plots of the LMM residuals showed acceptable linearity across the central quantiles for all 18 biomarkers after log transformation (Supplementary Figure S1). Although several biomarkers exhibited substantial departures from normality in their raw distributions, the applied log_10_ transformation markedly improved residual behavior, and no systematic deviations from normality were observed in the residual Q–Q plots. Residual-vs-predicted plots showed no pattern indicative of heteroscedasticity. No extreme values were detected (standardized residual >3 in any subject for any biomarker). The random-intercept specification adequately captured the within-subject correlation induced by the PRE–POST repeated measurements.

## 4. Discussion

### 4.1. Main Findings

This study examined potential sources of inter-individual variability in the hematological, biochemical, and endocrine responses previously characterized in Lidia bulls participating in Roping Bull celebrations [13]. Whereas our previous study primarily described the overall PRE–POST physiological changes associated with participation [13], the present investigation focused on whether the magnitude of these responses varied according to age, previous participation history, and sampling period. Significant post-event changes were observed in several biomarkers, including leukocyte counts, erythrocyte-related variables, muscle-associated enzymes, lactate, cortisol, and ACTH. The largest responses were detected for CK, LDH, and lactate, indicating a substantial physiological demand associated with participation in the event. The magnitude of these enzymatic and metabolic changes is consistent with the well-documented acute physiological response to strenuous exercise in cattle and other athletic species [13,15,16,17]. In contrast, most biomarkers showed limited influence of age, previous participation history, or sampling time, indicating that the overall physiological response was relatively consistent across the studied population. Nevertheless, selected Time × factor interactions suggested biomarker-specific inter-individual variability. Thus, the present study extends the previous physiological characterization [13] by identifying potential animal- and event-related factors associated with variability in the acute PRE–POST response, rather than providing a new descriptive characterization of the physiological variables themselves.

Unlike our previous study, which primarily described the overall physiological response associated with participation in Roping Bull celebrations, the present investigation focused on identifying factors that explain inter-individual variability in these responses. The use of mixed-effects models allowed evaluation of the influence of age, previous participation history, and event-related variables while accounting for repeated measurements, thereby extending the interpretation of the physiological data beyond simple pre- and post-event comparisons.

The present study should therefore be considered as an extension of our previous work rather than a repetition of it. While our earlier publication primarily described the acute physiological response of Lidia bulls participating in Roping Bull celebrations, the current investigation was specifically designed to identify factors associated with variability in those responses. By applying linear mixed-effects models to evaluate the influence of age, previous participation history, and event-related variables, the present study provides additional information regarding the determinants of physiological adaptation beyond the descriptive findings previously reported.

### 4.2. Physiological Response to Exercise and Stress

The marked increases in CK, LDH, and lactate observed after participation are consistent with the metabolic and muscular demands of prolonged high-intensity exercise. Similar responses have been reported in cattle exposed to strenuous physical activity, transport, and handling stress, where increased anaerobic metabolism and greater muscular workload lead to elevated circulating concentrations of these biomarkers [18,19,20]. The increase in lactate suggests an important contribution of anaerobic energy pathways during the event, as classically described for working muscles under fully aerobic or partially anaerobic conditions [21], whereas the elevation of CK and LDH reflects increased membrane permeability and muscular exertion [16,22,23]. Reference values for plasma biochemistry in athletic species such as Spanish purebred horses provide useful comparative benchmarks for these enzymatic responses [24], and CK and LDH elevations of similar magnitude have been associated with subsequent meat-quality deterioration in cattle [25].

Importantly, although these biomarkers increased significantly after participation, their interpretation should be approached cautiously. Elevated enzyme activities do not necessarily indicate clinically relevant muscle injury but rather reflect physiological responses commonly associated with intense exercise. Similar patterns of CK and LDH elevation without overt myopathy have been documented in working and transport-stressed cattle [26,27]. Additional studies, including post-event recovery monitoring, would be necessary to determine the duration and biological significance of these changes.

### 4.3. Activation of the Stress Response

Participation in the event was also associated with increased cortisol and ACTH concentrations, indicating activation of the hypothalamic–pituitary–adrenal (HPA) axis. This endocrine response is a well-recognized component of the physiological adaptation to both physical exercise and environmental challenges, as classically described for the general stress response [28,29] and shown across mammalian species exposed to extrinsic stressors [30] and for the integrative actions of glucocorticoids on metabolism, immunity, and behavior [31]. The endocrine response to competitive exercise has also been well documented in athletic horses [32,33] and in cattle subjected to transport and handling stressors [34,35]. In the present context, the observed hormonal changes likely reflect the combined influence of exercise, handling procedures, exposure to unfamiliar environments, and interaction with participants and spectators. The cortisol and ACTH concentrations measured after the event should be interpreted as an early post-event endocrine response. Because the POST sample was obtained within approximately 5 min after completion, circulating cortisol may not yet have reached its maximal post-stimulus concentration [32,33,34,35]. Consequently, the present measurements characterize the early endocrine response rather than the complete temporal profile or peak HPA-axis response.

The concurrent increase in leukocyte counts and neutrophils, together with the reduction in lymphocyte percentages, is consistent with the stress leukogram commonly reported in cattle experiencing acute stress and tissue trauma [36,37]. Comparable cortisol-driven leukocyte redistribution patterns have been observed in young beef bulls after transport [34] and in athletic horses before and after competitive jumping [38]. These hematological changes are generally attributed to glucocorticoid-mediated redistribution of circulating leukocyte populations rather than to inflammatory disease processes.

### 4.4. Effects of Age, Moment, and Previous Participation

One objective of this study was to determine whether physiological responses differed according to age or previous participation history. Overall, most biomarkers exhibited similar PRE-to-POST changes across groups. Although statistically significant interactions were detected for selected biomarkers, their effect sizes were generally smaller than those observed for the dominant PRE–POST Time effect. These findings therefore indicate statistical heterogeneity in the response rather than large biological differences among age or previous-participation groups. Accordingly, the interaction effects should be interpreted as evidence of modest modulation of the acute physiological response rather than as major determinants of physiological reactivity. The lack of large age-related differences observed in the present study is broadly consistent with previous reports describing relatively stable metabolic, enzymatic, and physiological responses to strenuous exercise in adult athletic animals [39,40]. Likewise, previous participation history had limited influence on the observed responses, suggesting that physiological reactivity to this particular event was not strongly modulated by the number of previous participations. Specifically, the Time × RACES interaction observed for WBC should be interpreted cautiously because differences among participation groups were partly attributable to baseline PRE values rather than to a clearly different magnitude of the PRE–POST change. Therefore, this finding does not provide strong evidence that previous participation modifies the acute leukocyte response or demonstrates physiological habituation. However, it remains possible that temperament may play a more substantial role when animals are evaluated using individual behavioral axes [41,42].

These findings suggest that the physiological demands associated with the event are relatively consistent among adult Lidia bulls within the age range evaluated. Temperament traits have been shown to influence stress reactivity and productivity in beef cattle [43], and previous work has highlighted the relevance of individual behavioral phenotypes for physiological responses to handling and transport [35,44]. Previous participation history should not be interpreted as a direct measure of physiological habituation. The RACES categories used in the present study provide a broad indicator of previous exposure, and the available range of experience may have been insufficient to detect progressive habituation effects. Because the present study was not specifically designed to assess training adaptation or behavioral habituation, future studies including animals with a wider and more detailed range of previous participation, including individuals with substantially greater exposure, would be valuable for testing this hypothesis directly.

The absence of a strong MOMENT effect should not be interpreted as evidence that environmental conditions were equivalent across events. Because ambient temperature and relative humidity were not systematically recorded, residual environmental variation among locations, dates, and years cannot be excluded. The inclusion of the sampling period as a fixed factor accounts only partially for this potential source of variability.

### 4.5. Implications for Animal Welfare

The present findings demonstrate that participation in Roping Bull celebrations is associated with measurable physiological responses involving metabolic, muscular, hematological, and endocrine systems. These responses indicate that the event represents a significant physiological challenge for the animals, and they are quantitatively comparable to those described for cattle during transport and lairage [19], for bulls during handling and restraint [27,44], and for horses during competitive exercise [24,32,33]. Together, these comparisons support the view that the magnitude of the acute physiological load imposed by the event is substantial and warrants explicit consideration in any welfare assessment framework [13,45,46,47].

Nevertheless, physiological activation should not be interpreted directly as evidence of impaired welfare. Stress-related responses constitute normal adaptive mechanisms that allow animals to cope with environmental and physical demands, as conceptualized in the biologically grounded framework of the stress response [28,29]. At the same time, the magnitude of the observed changes, together with the well-established link between stress reactivity, temperament, and subsequent performance [41,43], highlights the importance of continued evaluation of management practices aimed at minimizing unnecessary physiological burden.

Likewise, the observation that most biomarkers remained within published physiological reference intervals should be interpreted with caution. Clinical reference intervals are statistical descriptors derived from values observed in reference populations and do not represent thresholds of biological safety, acceptable welfare, or absence of adverse effects. Consequently, remaining within these intervals does not exclude the possibility that animals experienced a substantial physiological challenge during the event. Rather, the present findings indicate that the measured responses were compatible with those previously reported in healthy cattle exposed to acute exercise and handling stress, while recognizing that physiological biomarkers alone cannot determine the overall welfare status of the animals.

The results presented here provide objective data that may contribute to evidence-based discussions regarding animal management and welfare assessment in traditional bull-related events. However, welfare is a multidimensional concept encompassing physiological, behavioral, and health-related indicators and therefore cannot be fully evaluated using blood biomarkers alone. Future welfare-oriented studies should therefore combine physiological biomarkers with complementary animal-based indicators, including standardized behavioral assessments, avoidance responses, vocalization, heart rate and heart-rate variability, infrared thermography, injury assessment, and post-event recovery measures such as feed and water intake and restoration of baseline physiological values [27,29,43,45,46,47]. Heart rate and heart-rate variability may provide complementary information on autonomic responses to stress, while behavioral and recovery measures can contribute additional information that cannot be inferred from circulating biomarkers alone [27,47]. Such a multidimensional approach would provide a more comprehensive assessment of the physiological and behavioral consequences of participation in the event.

### 4.6. Limitations

Several limitations should be considered when interpreting the results. First, the study employed a PRE–POST observational design without a non-participating control group. Consequently, the relative contributions of exercise, transport, handling, restraint, and environmental factors cannot be distinguished, a limitation that is common in field studies of this type [19,27,44]. The PRE–POST design therefore allows characterization of the physiological changes associated with participation but does not permit causal attribution to exercise alone.

Second, although the duration of the Roping Bull activity was standardized at approximately 45 min, individual exercise intensity was not objectively quantified. Running speed, distance travelled, locomotor activity, and movement–rest patterns were not recorded. Therefore, differences in the magnitude of individual physical exertion could not be incorporated into the statistical models. Similarly, heart rate, respiratory rate, and other continuous cardiovascular or respiratory indicators were not recorded and therefore could not be incorporated into the physiological assessment. The absence of these measurements limits the characterization of the cardiovascular and respiratory components of the acute response.

Third, only acute PRE–POST responses were evaluated, and no information was available regarding post-event recovery dynamics or the time course of muscle-enzyme normalization. Such recovery patterns may extend beyond the immediate post-event period in exercising animals [15,16]. In addition, the POST sample was obtained within approximately 5 min after completion of the event and therefore represents an early post-event measurement rather than necessarily the peak or complete temporal endocrine and metabolic response.

Fourth, behavioral indicators and direct measures of the animal affective state were not assessed. Standardized behavioral or temperament assessments, together with physiological measurements, could provide complementary information for future welfare-oriented studies [35,41,42,43]. Accordingly, the physiological biomarkers evaluated here should not be interpreted in isolation as a direct measure of either preserved or compromised animal welfare.

Fifth, the events were conducted within the established seasonal and organizational context of the respective celebrations; however, ambient temperature and relative humidity were not systematically recorded at the event locations. Therefore, environmental conditions cannot be assumed to have been equivalent among locations, dates, or years, and residual environmental variation may have contributed to inter-individual or between-event variability in physiological responses. Although the sampling period (MOMENT; morning vs. afternoon) was included as a fixed effect in the statistical models, this factor accounts only partially for potential environmental variation. Future studies should incorporate standardized measurements of ambient temperature and relative humidity at the time of each event.

Sixth, detailed assay-specific information regarding intra-assay and inter-assay precision, limits of detection, and specific validation of the ACTH assay for bovine plasma was not available to the authors from the laboratory. These analytical characteristics could therefore not be independently reported or evaluated. Similarly, detailed documentation regarding species-specific calibration settings for the Cell-Dyn 3700 hematology analyzer was not available. The hematological results should therefore be interpreted in the context of the analytical procedures described in the Methods.

Seventh, of the 101 bulls included in the present study, 53 (52.4%) had also been included in a previous investigation [13], in which the physiological variables evaluated here had previously been reported. The present study should therefore be considered an analytical extension focused on potential sources of inter-individual variability rather than an entirely independent physiological dataset. The scientific objective differs from the previous descriptive characterization by evaluating whether the magnitude of the PRE–POST responses varies according to AGE, previous participation history (RACES), and sampling period (MOMENT). In addition, the sensitivity analysis based on change scores (POST–PRE) should be interpreted cautiously because the baseline PRE value is mathematically incorporated into the change score. Significant associations between PRE values and Δ therefore may partly reflect mathematical coupling and regression-to-the-mean effects rather than a purely biological relationship. For this reason, the change-score analysis was considered complementary, whereas the repeated-measures LMM remained the primary inferential framework.

Eighth, although information on genetic background or “encaste” was available, it was not included in the present statistical models because the study was specifically designed to evaluate the prespecified animal- and event-related factors AGE, previous participation history (RACES), and sampling period (MOMENT). Given the biological and behavioural diversity among Lidia lineages, genetic background may contribute to residual inter-individual variability in physiological responses. Future studies specifically designed to investigate genetic or lineage-related effects should incorporate “encaste” as an explanatory factor.

Finally, although the overall sample size was substantial for a field study of this type, some subgroup and interaction analyses may have had limited power to detect small effects. The magnitude and direction of selected interaction effects should therefore be interpreted cautiously, particularly where confidence intervals were wide. Future studies incorporating larger numbers of animals across age, previous participation, genetic background, environmental conditions, and repeated events would help to determine the consistency and biological relevance of these potential sources of variability.

## 5. Conclusions

Building upon our previous descriptive study, the present investigation demonstrates that the physiological responses of Lidia bulls participating in traditional Roping Bull celebrations are consistently influenced, but only to a limited extent, by animal- and event-related factors, indicating that the acute response pattern is relatively homogeneous across the studied population. Participation in Roping Bull celebrations was associated with a marked acute physiological response involving metabolic, muscular, hematological, and endocrine systems. The magnitude of these responses was generally more strongly related to the event itself than to age, previous participation history, or sampling period, although selected interactions indicated modest inter-individual variation. These findings extend previous descriptive work by identifying factors associated with variability in the acute physiological response. However, the present physiological measurements cannot by themselves determine whether animal welfare was preserved or compromised. Future studies integrating behavioral assessment, cardiovascular monitoring, environmental measurements, injury assessment, and post-event recovery are warranted to provide a more comprehensive evaluation of welfare during these traditional events.

## Figures and Tables

**Table 1:** Descriptive statistics of the 18 biomarkers at PRE and POST assessments (mean ± SD, or median [IQR] for non-normal distributions).

Biomarker	Unit	Transform	PRE	POST	Shapiro–Wilk *p* (PRE/POST)
WBC	10^3^/µL	log_10_	7.4 [6.3–9.3]	8.2 [6.8–10.3]	0.003/0.001
RBC	10^6^/µL	—	7.7 ± 0.9	8.18 ± 1.02	0.070/0.165
PLT	10^3^/µL	log_10_	292.0 [215.0–352.0]	311.0 [240.0–383.0]	0.001/0.011
HCT	%	log_10_	40.0 [34.0–43.0]	42.0 [37.0–45.0]	0.008/0.013
HGB	g/dL	log_10_	13.1 [11.2–14.1]	13.5 ± 1.9	0.026/0.348
MCV	fL	log_10_	51.0 [48.0–54.0]	50.0 [48.0–53.0]	0.003/0.023
MCH	pg	log_10_	17.0 [16.0–18.0]	17.0 [16.0–17.0]	<0.001/<0.001
MCHC	g/dL	log_10_	33.0 [32.0–33.0]	33.0 [32.0–34.0]	<0.001/<0.001
NEUT	%	—	45.5 ± 6.5	49.2 ± 6.6	0.116/0.068
EOS	%	log_10_	2.0 [1.0–3.0]	2.0 [1.0–3.0]	<0.001/<0.001
BASO	%	log_10_	0.0 [0.0–0.0]	0.0 ± 0.0	<0.001/—
LYM	%	log_10_	48.0 [46.0–52.0]	47.0 [43.0–50.0]	<0.001/0.009
MON	%	log_10_	2.0 [1.0–4.0]	2.0 [1.0–3.0]	<0.001/<0.001
LDH	IU/L	log_10_	413.0 [299.0–495.0]	653.1 ± 181.3	<0.001/0.060
CK	IU/L	log_10_	103.0 [86.0–151.0]	170.0 [152.0–266.0]	<0.001/<0.001
LAC	mg/dL	log_10_	24.3 ± 5.5	32.6 [26.3–36.9]	0.391/<0.001
CORT	µg/dL	log_10_	3.7 [2.9–6.0]	4.8 [3.5–7.6]	<0.001/<0.001
ACTH	pg/mL	log_10_	16.0 [11.0–19.0]	19.0 [14.0–23.0]	<0.001/<0.001

Footnote: Values are reported as mean ± SD for normally distributed biomarkers and as median [interquartile range, IQR] for those with significant deviations from normality (Shapiro–Wilk *p* < 0.05) in PRE and/or POST assessments. Abbreviations: WBC = white blood cells; RBC = red blood cells; PLT = platelets; HCT = hematocrit; HGB = hemoglobin; MCV = mean corpuscular volume; MCH = mean corpuscular hemoglobin; MCHC = mean corpuscular hemoglobin concentration; NEUT = neutrophils; EOS = eosinophils; BASO = basophils; LYM = lymphocytes; MON = monocytes; LDH = lactate dehydrogenase; CK = creatine kinase; LAC = lactate; CORT = cortisol; ACTH = adrenocorticotropic hormone. Lactate concentrations are presented in mg/dL; to convert to mmol/L, values should be multiplied by 0.111.

**Table 2:** Normality assessment and log_10_(x + 1) transformation decisions based on the Shapiro–Wilk test.

Transformation	Biomarkers	Justification
Applied (16)	WBC, PLT, HCT, HGB, MCV, MCH, MCHC, EOS, BASO, LYM, MON, LDH, CK, LAC, CORT, ACTH	Shapiro–Wilk *p* < 0.05 in PRE and/or POST; visual Q–Q plot showed departure from the diagonal.
Not applied (2)	RBC, NEUT	Shapiro–Wilk *p* ≥ 0.05 in both PRE and POST; visual inspection of Q–Q plots showed acceptable linearity.

Footnote: The Shapiro–Wilk test was applied separately to PRE and POST distributions of each biomarker (*n* = 101 each). Biomarkers were log_10_(x + 1)-transformed when normality was rejected (*p* < 0.05) in either PRE or POST. The transformation preserves the sign and zero of the original values while stabilizing variance and reducing right-skewness. RBC and NEUT were not transformed because their distributions did not significantly deviate from normality in either time point.

## Data Availability

The original contributions presented in the study are included in the article, and further inquiries can be directed to the corresponding author.

## Supplementary Materials

The following supporting information can be downloaded at: https://www.mdpi.com/article/10.3390/ani16172739/s1, Table S1a. Sensitivity analysis using a Generalized Linear Model (GZLM) on change scores (Δ = POST−PRE) for the 18 biomarkers. The table reports the F-statistic and Bonferroni-adjusted *p*-value for the PRE value (as a covariate) and the three main effects (RACES, AGE, MOMENT). The dependent variable Δ was computed on the same transformed scale as the LMM (log_10_ for 16 of 18 biomarkers); Table S1b. Two-way interaction effects (RACES × AGE, RACES × MOMENT, AGE × MOMENT) from the GZLM sensitivity analysis on Δ. F-statistic and Bonferroni-adjusted *p*-values are reported. None of the two-way interactions remained significant after correction, indicating that the PRE–POST change is homogeneous across all factor combinations. Significance: * *p* < 0.05, ** *p* < 0.01, *** *p* < 0.001, ns = non-significant; Table S1c. Three-way interaction effect (RACES × AGE × MOMENT) from the GZLM sensitivity analysis on Δ. F-statistic and Bonferroni-adjusted *p*-values are reported. Only one of 18 biomarkers (BASO, which had essentially no variance in POST) showed a nominally significant three-way interaction, but this is not interpretable and should be disregarded. Significance: * *p* < 0.05, ** *p* < 0.01, *** *p* < 0.001, ns = non-significant; Figure S1. Q–Q plots of the residuals from the linear mixed-effects model for the 9 biomarkers with the largest Time effect (in terms of partial eta-squared). Deviation from the diagonal indicates departure from the normality assumption for the residuals. The Shapiro–Wilk *p*-value for the residuals is annotated in each panel.

## Abbreviations

The following abbreviations are used in this manuscript:

ACTH	Adrenocorticotropic hormone or adrenocorticotropin
ADP	Adenosine diphosphate
ATP	Adenosine triphosphate
CK	Creatine kinase
HCT	Hematocrit
HGB	Hemoglobin
LBBSB	Lidia Bovine Breed Stud Book
LDH	Lactate dehydrogenase
MCH	Mean corpuscular hemoglobin
MCHC	Mean corpuscular hemoglobin concentration
MCV	Mean corpuscular volume
NAD	Nicotinamide adenine dinucleotide
NADH	Nicotinamide adenine dinucleotide hydrogenase
PLT	Platelets
POST	After
PRE	Before
RBC	Red blood cells
SD	Standard deviation
WBC	White blood cells
