# Supplementary material for: Acute Physiological Response Variability in Lidia Bulls Following Roping Bull Celebrations: Effects of Age, Previous Participation, and Sampling Period
Source: Animals (Basel). 2026 Sep 2;16(17):2739. doi: 10.3390/ani16172739 (PMC13564484; doi:10.3390/ani16172739)
Supplement: Supplementary file 1 [file animals-16-02739-s001.zip › animals-4444494-supplementary.pdf]

Supplementary Materials

The following supplementary materials provide additional methodological details and robustness checks for the analyses presented in the main text.

Supplementary Table S1a. Sensitivity analysis using a Generalized Linear Model (GZLM) on change scores (Δ = POST − PRE) for the 18 biomarkers. The table reports the F-statistic and Bonferroni-adjusted p-value for the PRE value (as a covariate) and the three main effects (RACES, AGE, MOMENT). The dependent variable Δ was computed on the same transformed scale as the LMM (log_{10} for 16 of 18 biomarkers).

Biomarker	log_{10}	PRE F	PRE p	RACES F	RACES p	AGE F	AGE p	MOMENT F	MOMENT p
WBC	yes	10.48	0.031*	0.45	1.000ns	5.39	0.114ns	0.11	1.000ns
RBC	no	2.90	1.000ns	0.73	1.000ns	0.94	1.000ns	0.00	1.000ns
PLT	yes	7.46	0.139ns	0.09	1.000ns	2.01	1.000ns	0.00	1.000ns
HCT	yes	11.64	0.018*	0.87	1.000ns	2.67	1.000ns	0.09	1.000ns
HGB	yes	9.27	0.056ns	0.99	1.000ns	1.21	1.000ns	0.86	1.000ns
MCV	yes	9.11	0.061ns	0.36	1.000ns	4.69	0.212ns	0.51	1.000ns
MCH	yes	14.78	0.004**	0.69	1.000ns	0.32	1.000ns	1.10	1.000ns
MCHC	yes	12.80	0.011*	0.22	1.000ns	0.61	1.000ns	6.31	0.251ns
NEUT	no	20.31	0.000***	0.12	1.000ns	0.57	1.000ns	0.09	1.000ns
EOS	yes	35.47	0.000***	0.10	1.000ns	1.35	1.000ns	0.22	1.000ns
BASO	yes	—	0.000***	3.03	0.966ns	6.72	0.036*	2.70	1.000ns
LYM	yes	9.01	0.064ns	0.33	1.000ns	0.27	1.000ns	0.03	1.000ns
MON	yes	25.77	0.000***	0.29	1.000ns	2.36	1.000ns	0.39	1.000ns
LDH	yes	30.10	0.000***	1.24	1.000ns	0.14	1.000ns	0.06	1.000ns
CK	yes	19.21	0.001***	0.18	1.000ns	0.15	1.000ns	0.80	1.000ns
LAC	yes	7.40	0.144ns	1.61	1.000ns	0.96	1.000ns	0.17	1.000ns
CORT	yes	4.22	0.776ns	0.24	1.000ns	0.06	1.000ns	0.01	1.000ns
ACTH	yes	4.15	0.808ns	0.07	1.000ns	0.78	1.000ns	0.06	1.000ns

*Footnote: Sensitivity analysis using a Generalized Linear Model (GZLM) on change scores (Δ = POST − PRE). The dependent variable Δ was computed on the same transformed scale as the LMM (log_{10} for 16 of 18 biomarkers). The PRE value was log_{10}-transformed for biomarkers with significant non-normality and entered as a covariate. RACES, AGE, and MOMENT were modelled as fixed factors. F-statistics and Bonferroni-adjusted p-values (across 18 outcomes) are reported. Significance: * $p < 0.05$, ** $p < 0.01$, *** $p < 0.001$, ns = non-significant.*

Supplementary Table S1b. Two-way interaction effects (RACES × AGE, RACES × MOMENT, AGE × MOMENT) from the GZLM sensitivity analysis on Δ. F-statistic and Bonferroni-adjusted p-values are reported. None of the two-way interactions remained significant after correction, indicating that the PRE→POST change is homogeneous across all factor combinations. Significance: * $p < 0.05$, ** $p < 0.01$, *** $p < 0.001$, ns = non-significant.

Biomarker	log_{10}	R×A F	R×A p	R×M F	R×M p	A×M F	A×M p
WBC	yes	4.27	0.062ns	0.27	1.000ns	3.40	0.684ns
RBC	no	1.46	1.000ns	1.51	1.000ns	0.38	1.000ns
PLT	yes	0.87	1.000ns	0.23	1.000ns	1.99	1.000ns
HCT	yes	2.64	0.709ns	1.23	1.000ns	1.12	1.000ns
HGB	yes	1.15	1.000ns	1.49	1.000ns	0.10	1.000ns

MCV	yes	2.45	0.939ns	0.20	1.000ns	2.47	1.000ns
MCH	yes	1.49	1.000ns	0.60	1.000ns	0.02	1.000ns
MCHC	yes	1.74	1.000ns	1.62	1.000ns	2.06	1.000ns
NEUT	no	0.57	1.000ns	0.31	1.000ns	0.27	1.000ns
EOS	yes	1.89	1.000ns	0.49	1.000ns	2.05	1.000ns
BASO	yes	1.70	1.000ns	1.11	1.000ns	19.72	0.000***
LYM	yes	0.43	1.000ns	0.10	1.000ns	0.02	1.000ns
MON	yes	0.06	1.000ns	0.03	1.000ns	0.98	1.000ns
LDH	yes	0.46	1.000ns	0.89	1.000ns	0.15	1.000ns
CK	yes	0.46	1.000ns	0.43	1.000ns	0.43	1.000ns
LAC	yes	1.49	1.000ns	2.16	1.000ns	0.37	1.000ns
CORT	yes	1.95	1.000ns	0.13	1.000ns	0.46	1.000ns
ACTH	yes	0.86	1.000ns	1.49	1.000ns	0.57	1.000ns

*Footnote: Two-way interaction effects (RACES × AGE, RACES × MOMENT, AGE × MOMENT) from the GZLM sensitivity analysis on change scores (Δ = POST − PRE). F-statistics and Bonferroni-adjusted p-values are reported. None of the two-way interactions remained significant after correction, indicating that the PRE–POST change is homogeneous across all factor combinations. Significance: * p < 0.05, ** p < 0.01, *** p < 0.001, ns = non-significant.*

Supplementary Table S1c. Three-way interaction effect (RACES × AGE × MOMENT) from the GZLM sensitivity analysis on Δ. F-statistic and Bonferroni-adjusted p-values are reported. Only one of 18 biomarkers (BASO, which had essentially no variance in POST) showed a nominally significant three-way interaction, but this is not interpretable and should be disregarded. Significance: * p < 0.05, ** p < 0.01, *** p < 0.001, ns = non-significant.

Biomarker	log_{10}	R×A×M F	R×A×M p	Interpretation
WBC	yes	1.90	1.000ns	Homogeneous
RBC	no	0.24	1.000ns	Homogeneous
PLT	yes	1.04	1.000ns	Homogeneous
HCT	yes	0.43	1.000ns	Homogeneous
HGB	yes	0.31	1.000ns	Homogeneous
MCV	yes	0.82	1.000ns	Homogeneous
MCH	yes	0.97	1.000ns	Homogeneous
MCHC	yes	0.25	1.000ns	Homogeneous
NEUT	no	0.70	1.000ns	Homogeneous
EOS	yes	2.26	1.000ns	Homogeneous
BASO	yes	3.79	0.126ns	Homogeneous
LYM	yes	0.40	1.000ns	Homogeneous
MON	yes	0.29	1.000ns	Homogeneous
LDH	yes	0.81	1.000ns	Homogeneous
CK	yes	0.44	1.000ns	Homogeneous
LAC	yes	0.54	1.000ns	Homogeneous
CORT	yes	2.13	1.000ns	Homogeneous
ACTH	yes	2.41	1.000ns	Homogeneous

*Footnote: Three-way interaction effect (RACES × AGE × MOMENT) from the GZLM sensitivity analysis on change scores (Δ = POST − PRE). F-statistic and Bonferroni-adjusted p-value are reported. Only one of 18 biomarkers (BASO, which had essentially no variance in POST) showed a nominally significant three-way interaction; this is a statistical artefact of the near-zero denominator and should be disregarded. Significance: * p < 0.05, ** p < 0.01, *** p < 0.001, ns = non-significant.*

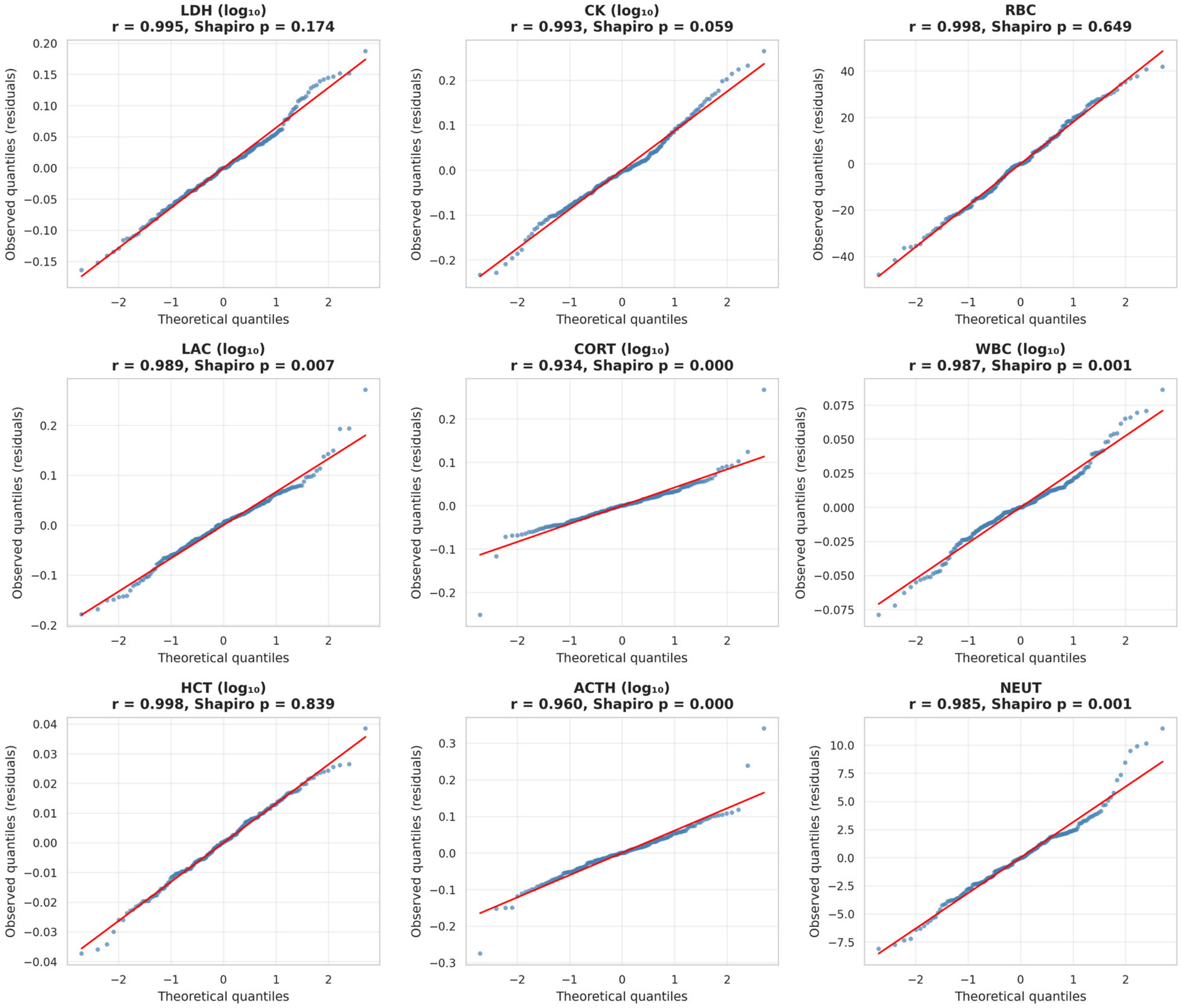

Supplementary Figure S1. Q–Q plots of the residuals from the linear mixed-effects model for the 9 biomarkers with the largest Time effect (in terms of partial eta-squared). Deviation from the diagonal indicates departure from the normality assumption for the residuals. The Shapiro–Wilk p-value for the residuals is annotated in each panel.
